# Isolation and Proliferation of Spermatogonial Cells from Ghezel Sheep

**Published:** 2018

**Authors:** Babak Qasemi-Panahi, Mansoureh Movahedin, Gholamali Moghaddam, Parviz Tajik, Mortaza Koruji, Javad Ashrafi-Helan, Seyed Abbas Rafat

**Affiliations:** 1. Department of Animal Science, Faculty of Agriculture, University of Tabriz, Tabriz, Iran; 2. Department of Anatomy, Faculty of Medical Science, University of Tarbiat Modares, Tehran, Iran; 3. Department of Theriogenology, Faculty of Veterinary Medicine, University of Tehran, Tehran, Iran; 4. Department of Anatomy, Faculty of Medical Science, Iran University of Medical Sciences, Tehran, Iran; 5. Department of Pathobiology, Faculty of Veterinary Medicine, University of Tabriz, Tabriz, Iran

**Keywords:** Ghezel sheep, Isolation, Spermatogonia

## Abstract

**Background:**

Sheep industry has taken steps toward transforming itself into a more efficient and competitive field. There are many varieties of sheep breeds in the world that each of them serves a useful purpose in the economies of different civilizations. Ghezel sheep is one of the Iranian important breeds that are raised for meat, milk and wool. Field of spermatogonial cell technologies provides tools for genetic improvement of sheep herd and multiple opportunities for research. Spermatogonial cells are the only stem cells capable of transmitting genetic information to future generations.

**Methods:**

This study was designed to extend the technique of isolation and *in vitro* proliferation of spermatogonial cells in Ghezel sheep.

**Results:**

Isolated cells were characterized further by using specific markers for type A spermatogonia, including PLZF. Also, sertoli cells were characterized by vimentin which is a specific marker for sertoli cells. After 10 days of co-culture, viability rates of the cells was above 94.7%, but after the freezing process the viability rates were 74 percent.

**Conclusion:**

In this study, a standard method for isolation and *in vitro* proliferation of spermatogonial stem cells in Ghezel sheep was developed.

## Introduction

Ghezel sheep are raised in north-west of Iran and north east of Turkey. This breed is native, fat-tailed and large-sized. Birth weight, weaning weight, weight at six months, average daily gain from birth to weaning and average daily gain from weaning to six months were 4.47, 20.88, 33.14 *Kg*, 0.184 and 0.134 *gr,* respectively^1^.

The economic importance of sheep industry has led to the progress of new technologies such as cell culture, In Vitro Fertilization (IVF) and Intracytoplasmic Sperm Injection (ICSI) technique. Spermatogonial Stem Cell (SSC) technology in sheep has been more limited. SSCs have the ability to transmit genetic information to future generation, so these cells could be used in the generation of transgenic animals^2^. Advances in genomic editing, mainly the CRISPR-Cas9 system, are likely to permit genomic alteration of SSCs^3^. Also, SSC technology is a new tool for studying spermatogenesis^4^.

SSCs can be isolated, cultured and cryopreserved in mouse^5,6–9^, bovine^10,11^, goat^12^, pig^13^ and sheep^14^. But the ability to manipulate them in different species is unique. Therefore, this study was designed to establish the technique of spermatogonial isolation, proliferation and freezing protocol in sheep.

For creation of transgenic animals, establishment of this technology in specific breeds of sheep is important. The purpose of this study was to adapt spermatogonial cell isolation, co-culture them with sertoli cells and proliferate them in Ghezel breed sheep.

## Materials and Methods

### Animal

In this survey, two months old male lambs (Ghezel breed) were used. The research was conducted in accordance with the National Research Council guidelines. Lambs were sedated with 0.1 *mg/kg* IM administration of xylazine. Also, each lamb received a 3-*ml* local injection of 2% lidocaine 20 *min* before surgery. Respiration rate, heart rate, and rectal temperature were monitored before and after recovery from analgesia. Lambs were given flunixin meglumine (2.2 *mg/kg* of body weight, *IM*) for 3 days after surgery.

### Germ cell collection

A testis biopsy was taken from the right testis of all lambs. Testicular germ cell collection was done according to Qasemi-Panahi *et al’s* instructions^15^. Briefly, testis pieces were floated in DMEM containing 1 *mg/ml* collagenase type IV, 1 *mg/ml* trypsin, 1 *mg/ml* hyaluronidase type II and then incubated at 37°*C* for 40 *min*. All the enzymes and culture medium were purchased from Sigma (Sigma-Aldrich, St. Louis, MO, USA). After washing in DMEM, seminiferous tubules were entered in to secondary digestion process, with the same enzymes for 20 *min* at 37°*C*. To achieve favorite cell population, cellular suspension was centrifuged at 30 *g* for 2 *min*. The cells were then filtered through a nylon mesh with 60 *mm* pore size. The cells were pelleted and subjected to co-culture of spermatogonial and sertoli cells for 10 days.

Cells were cultured in DMEM supplemented with 10% Fetal Bovine Serum (FBS) (Gibco, Gaithersburg, MD, USA), 100 *IU/ml* penicillin and 100 *mg/ml* streptomycin for 10 days when the cells reached to about 80% confluency. Colonization began from day 4 after co-culture. The size and number of colonies were measured during the culture for 2 weeks but not mentioned in this paper.

### SSCs and sertoli cell identification

For identification of SSCs and sertoli cells in sheep, immunocytochemistry staining (ICC) was used. According to Bahadorani *et al*, as a specific marker of SSCs, anti-PLZF was used in this study^16^. Eight-well glass slides (Marienfeld, Germany) were used in which 1–2×10^4^ cells were grown in each well. The cells were incubated overnight in a humidified incubator followed by washing with pre-warmed medium. After being dried for 15 *min*, cells were fixed and permeabilized in acetone for 2 *min* at −20°*C* and kept at 4°*C* for 30 *min* until slides were completely dried. Slides were then washed three times (3×3 *min*) with Tris-buffered saline, pH=7.4 containing 5% Bovine Serum Albumin (TBS/BSA).

The obtained colonies of SSCs were immunocytochemically stained with anti-PLZF. Briefly, goat anti-PLZF (1:100, Santa Cruz Biotechnology, USA) was applied for 60 *min* at room temperature. After being washed, mouse anti-goat IgG (1:50, PLZF TexasRed) was added and incubation was further continued for 45 *min* at room temperature. After washing with TBS/BSA, the nuclei were counterstained by 4′,6-diamidi-no-2-phenylindole dihydrochloride (DAPI; Calbiochem, Nottingham, UK) at 0.1 *μg/ml* for 5 *min*, then the slides were washed, mounted in PBS-glycerol 90%, and examined under a fluorescence microscope (Olympus, Tokyo, Japan).

The obtained colonies of SSCs were immunocytochemically stained with anti Oct-4. Briefly, anti Oct-4 (Abcam) diluted in TBS/BSA was applied over slides. After 60 *min*, washing of slide was done. FITC conjugated donkey polyclonal secondary antibody to Goat IgG was added and incubation was further continued for 45 *min* at room temperature. After washing, it was mounted in PBS-glycerol 90%, and examined under a fluorescence microscope (Olympus, Tokyo, Japan).

Vimentin was detected in sertoli cells by the procedure which was described by Tajik *et al*^17^. Briefly, anti-vimentin (Abcam, Cambridge, UK) optimally diluted in TBS/BSA (2 *μg/ml*) was applied over slides for 60 *min* at room temperature. After being washed, Fluorescein Isothiocyanate (FITC)-conjugated sheep anti-mouse Ig was diluted in TBS/BSA at a ratio of 1:50 and incubation was further continued for 45 *min* at room temperature. Following the first washing with TBS/BSA, slide was exposed to 7-Amino-actinomycin D (7AAD) 5 *μg/ml* for 5 *min*. It was then washed and mounted in PBS-glycerol 90% and examined under a fluorescence microscope (Olympus, Tokyo, Japan).

### Cryopreservation

After 10 days of co-culture, spermatogonial and sertoli cells were cryopreserved according to Izadyar *et al’s* instructions with some modification^18^. The freezing media were based on DMEM supplemented with 90% (*v/v*) FBS, 1.4 *M* DMSO and 0.07 *M* sucrose. For freezing, cryovials (Nunc, Denmark) were placed in a polystyrene container at −80°*C* for at least 1 day and then plunged into liquid nitrogen. Thawing process was done at 38°*C* water bath for 2 *min*. A sample was taken for viability assessment.

## Results

**Figure 1.**
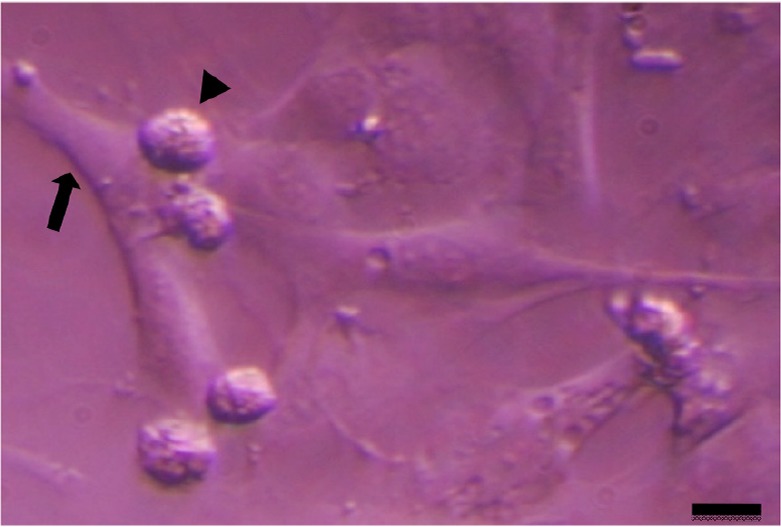
Sertoli cell (arrow) and spermatogonia (arrow head) of sheep. Scale bars represent 15 *μm*.

**Figure 2.**
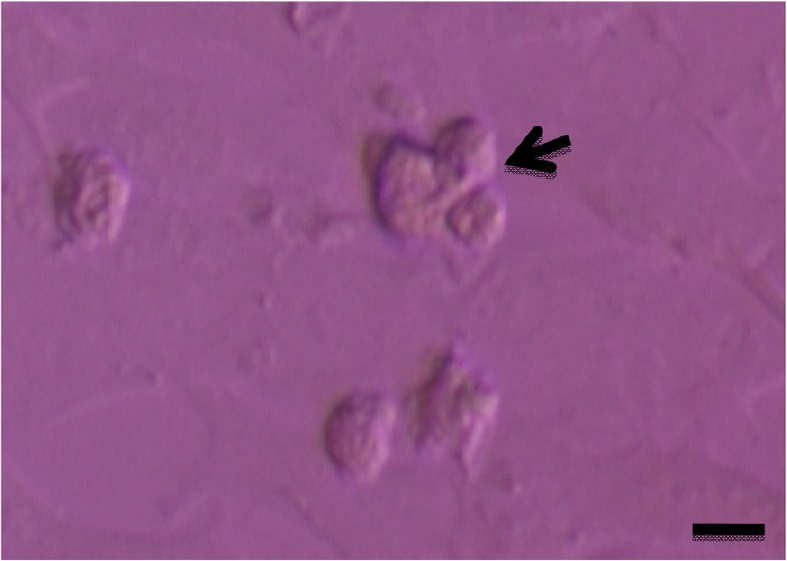
Cell clumps of sheep spermatogonia (arrow). Scale bars represent 15 *μm.*

**Figure 3.**
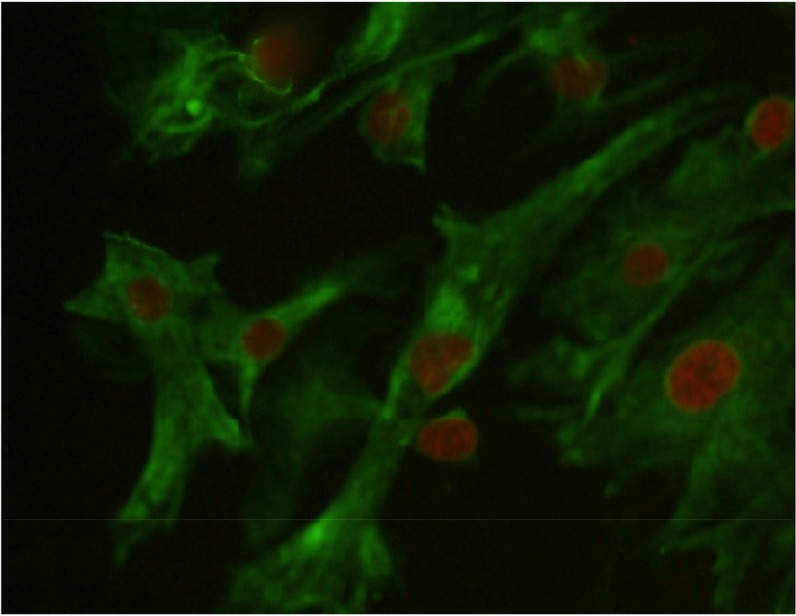
Vimentin was detected in the feeder monolayer cells (Vimentin-specific monoclonal antibodies). Nuclei were counterstained with 7AAD, (×40).

**Figure 4.**
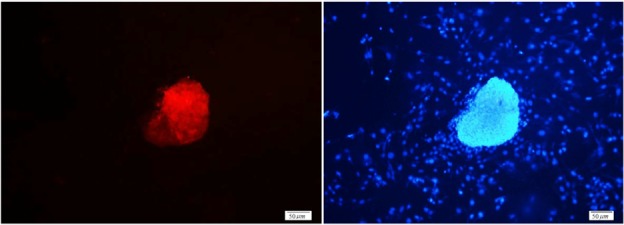
PLZF immunocytochemical staining of sheep spermatogonial stem cells. Nuclei were counterstained with DAPI (×20).

**Figure 5.**
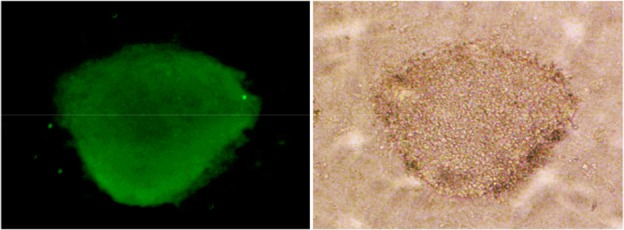
Oct-4 immunocytochemical staining of sheep spermatogonial stem cells (×40).

Testicular cells were isolated from testis samples of two months old lambs and co-cultured with sertoli cells. The sertoli cells which proliferated and created a monolayer of feeder cells and spermatogonia cells were colonized. Type A spermatogonia were round cells (Figure 1) and small colonies of these cells appeared four days after co-culture (Figure 2). Vimentin was detected in this monolayer cell (Figure 3). PLZF which is a molecular marker for SSCs was detected in the obtained round cells (Figure 4). Also, Oct-4 was detected in sheep SSCs (Figure 5) that is a specific marker for SSCs. The viability rate of the cells before and after freezing were 90.3 and 71%, respectively.

## Discussion

Ghezel sheep originated in northwestern Iran and northeastern Turkey. This region in Iran is known as Azarbayjan and is typified by dry, cold mountain weather^1,19^. Because of economic importance, the new biotechnological methods are applied in this industry. SSC technology in sheep has been more limited. These cells are the only cells in adults capable of transmitting genetic information to future generations^20^. Also, SSC technology with genetic engineering might be used to impede paternal transmission of genomic diseases^3^.

The number of SSCs in seminiferous tubules is 0.3%^21^. For cell handling, the culture and proliferation of these cells are necessary. There are many researches about the isolation and culture of spermatogonial cells in rodent, bovine, ovine and other species^4,17,21^. But, there are many differences among species of animals and isolation, culture and freezing methods for different species are needed to be set up. In this study, after one week of co-culture, the colonies of spermatogonial cells were appeared. Medium was changed every 3 days and colony diameter was growing slowly. This finding is in agreement with the reports by Koruji *et al* in rodents^25^. PLZF was detected in ovine spermatogonial cells which was in line with Costoya *et al’s*^22^, Phillips *et al’s*^23^ and Ketkar *et al’s*^24^ findings. In the testis, PLZF expression is restricted to As, Apr and Aal undifferentiated spermatogonia, including SSCs^25^. PLZF’s role in spermatogonia may be the maintenance of an undifferentiated state^26^, similar to the role suggested for PLZF in haematopoietic precursor cells^27^.

In other researches, Oct-4 was used as a specific marker for identification of bovine spermatogonial stem cells^15,28^. Similarly, in the present study, sheep spermatogonial stem cells were positive for anti Oct-4 immunoflourscent staining.

In addition, the presence of vimentin was analyzed in sertoli cells of lamb testes, using specific antibodies and immunocytochemistry staining. Vimentin was prominently present in sertoli cells in all cases investigated. These results are in line with Vogl *et al’s*^29^, Aumuller *et al’s*^30^ and Rogatsch *et al’s*^31^ findings. According to Wayne Vogl *et al*, unlike most other epithelia where the intermediate filaments are of keratin type, intermediate filaments in mature sertoli cells are of the vimentin type^32^. Vimentin filaments play an important role in the adaptation of sertoli cells to the varying configurations of neighboring cells during spermatogenesis as well as under pathological conditions^30^.

Due to diminishing cell recovery following the freeze/thaw procedure, the viability rate of frozen/thawed cells was lower than freshly isolated cells. Reduced cell recovery following the freeze/thaw procedure was also reported by others^6^.

SSCs’s colony number declined during the 2^nd^ and 3^rd^ weeks of culturing, probably due to apoptosis or detachment of differentiating germ cells^11^. So, for long term studies, establishment of a freezing method for long term preservation of these cells is needed. It seems that freezing medium containing 90% FBS and 10% DMSO is suitable for freezing procedure.

## Conclusion

The field of SSC technologies provides the tools for genetic improvement of sheep herds and multiple opportunities for research. Despite all the challenges that the development of SSC technologies raise, still many questions are open as how sheep work in a biotechnological setting. Progress in the field of sheep reproduction biotechnology will probably derive new applications not only for use in sheep but also in other ruminants, which are of great importance to the food production chains of many developing countries. In this study, a standard method for isolation and *in vitro* proliferation of SSCs in Ghezel sheep was developed.
